# A case report of neonatal incontinentia pigmenti complicated by severe cerebrovascular lesions in one of the male monozygotic twins

**DOI:** 10.3389/fped.2024.1338054

**Published:** 2024-05-21

**Authors:** Xiaofeng Lin, Wei Zhang, Ping Zhou

**Affiliations:** ^1^ Department of Neonatology, Shenzhen Baoan Women’s and Children’s Hospital, Shenzhen, China; ^2^ Department of Research and Development, BGI Genomics, Shenzhen, China

**Keywords:** monozygotic twins, neonatal, incontinentia pigmenti, cerebrovascular lesions, IKBKG gene

## Abstract

**Background:**

This article reports a case of neonatal incontinentia pigmenti onset in only one male monozygotic twin with characteristic skin lesions after birth followed by severe cerebrovascular lesions.

**Case presentation:**

A male infant, the first of monozygotic twins, was born with multiple yellow pustules all over his body, repeated new herpes at different sites during the course of the disease, aggravated by fusion, warty crusts, and hyperpigmentation; biopsy pathology suggested eosinophilic spongiform edema of the skin. Peripheral blood eosinophils were significantly elevated, and brain magnetic resonance imaging revealed diffuse multiple cystic and lamellar abnormal signal areas in the left frontal and parietal lobes. On day 30, the infant showed neurological symptoms, such as poor response and apnea, and an emergency cranial computed tomography scan revealed abnormal changes in the left cerebral hemisphere and bilateral cerebellum. After admission, he was given a potassium permanganate bath and topical mupirocin for 1 month, and the skin abnormalities improved. He was treated with mechanical ventilation and vasoactive drugs for 2 days after the cerebrovascular accident, and died the same day after the parents chose hospice care. No deletion variants or point mutations were detected in subsequent genetic tests, and chromosomal copy number variation tests revealed different degrees of chimeric duplications and deletions in different regions of chromosomes Y and 3. The parents were healthy, and his twin brother had normal growth and development with no abnormalities at multiple follow-up visits.

**Conclusion:**

Neonatal incontinentia pigmenti in only one male monozygotic twin is extremely rare and the genetic diagnosis is challenging. Awareness of the combined cerebrovascular lesions needs to be enhanced, and potential prevention and treatment methods need to be explored to improve the prognosis.

## 1 Introduction

Incontinentia pigmenti (IP), also known as Bloch–Sulzberger syndrome, is a disorder caused by mutations in the inhibitor of the nuclear factor kappa B kinase regulatory subunit gamma (IKBKG) gene that manifests primarily with skin damage in a characteristic four-stage sequence along the Blaschko line and may affect the hair, teeth, nails, eyes, and central nervous system (Online Mendelian Inheritance in Man, OMIM: 308300) (1). Its prevalence is approximately 1.2/1,000,000 and the mode of inheritance is X-linked dominant (2). Patients with IP are mostly female and male fetuses usually die *in utero*. Occasionally, male infants survive due to somatic mosaicism or a 47,XXY karyotype. No male monozygotic twins have been reported to develop IP alone or simultaneously (3). We recently treated an extremely rare case of neonatal IP in only one male monozygotic twin, which is reported below.

## 2 Case presentation

### 2.1 General information

**Figure 1:**
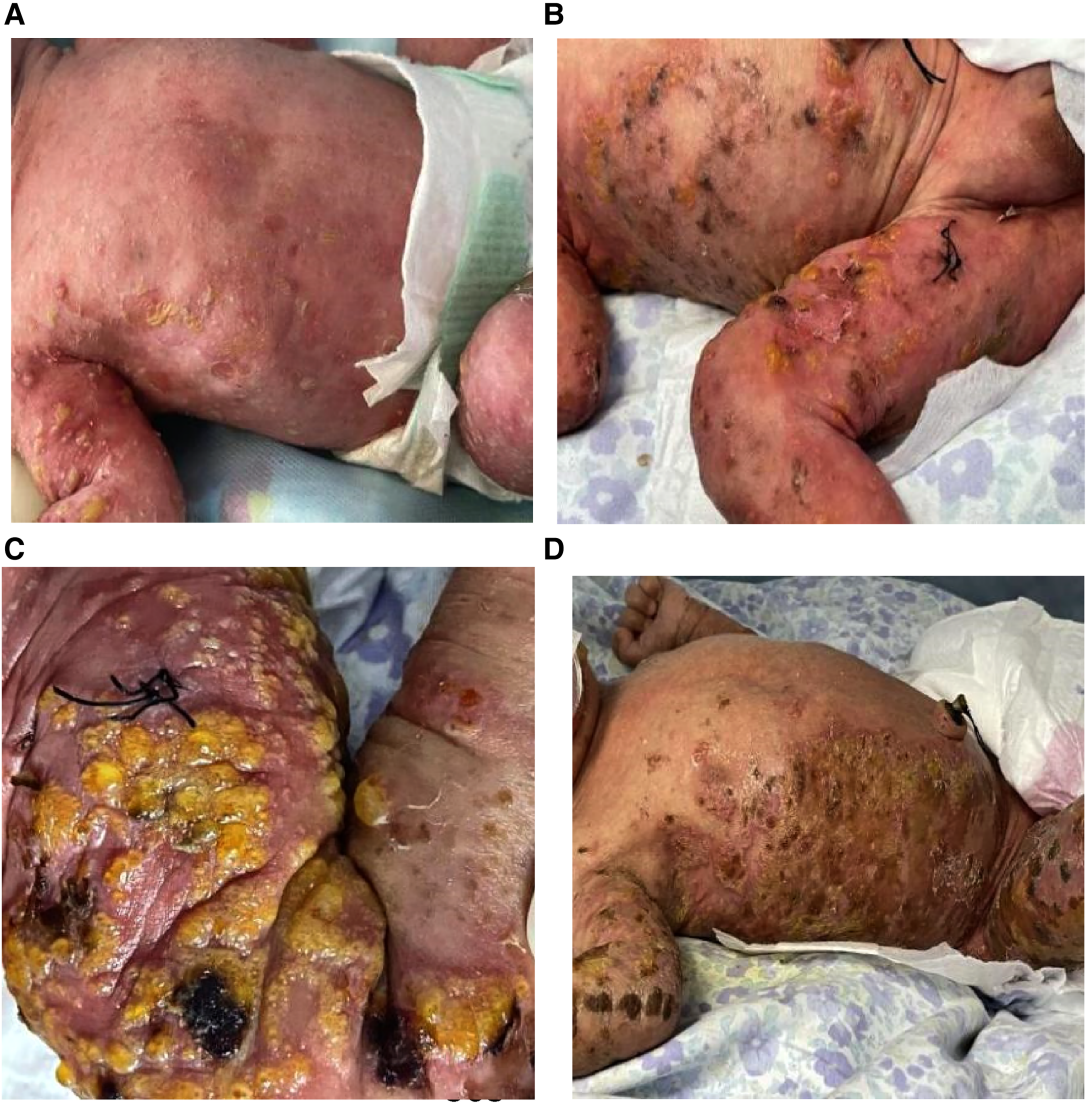
Manifestations of skin lesions at different stages after birth. (**A**) Corn to mung bean sized yellow pustules, erythema on day 1; (**B**) the original herpes fused into patches, partially pigmented, alternating with the newly appearing herpes; and (**C,D**) warty crusting and hyperpigmentation.

A male infant of Han ethnicity, G1P1, the first of monozygotic twins, with a gestational age of 36^+2^ weeks and birth weight of 2,120 g, was delivered by cesarean section in another hospital due to twin pregnancy. The twins were conceived naturally, with prenatal use of magnesium sulfate and a full course of steroids. The amniotic fluid, umbilical cord, placenta, and Apgar score were normal. Herpes was found scattered all over the body after birth, and the infant was then transferred to our hospital with herpes of unknown cause and preterm small for gestational age. His parents are healthy, not consanguineous, and denied any history of genetic disease. A physical examination showed stable vital signs and good mental response. Scattered yellow pustules the size of corn to green beans were seen on the extremities, trunk, axillae, and groin, with cloudy fluid and semilunar accumulation of pus, thin loose walls, and partial rupture with superficial erosion and a yellowish scab on top (Figure 1A). No abnormal findings were found during other systemic physical examinations.

### 2.2 Diagnostic testing

#### 2.2.1 Laboratory tests and imaging examinations

**Figure 2:**
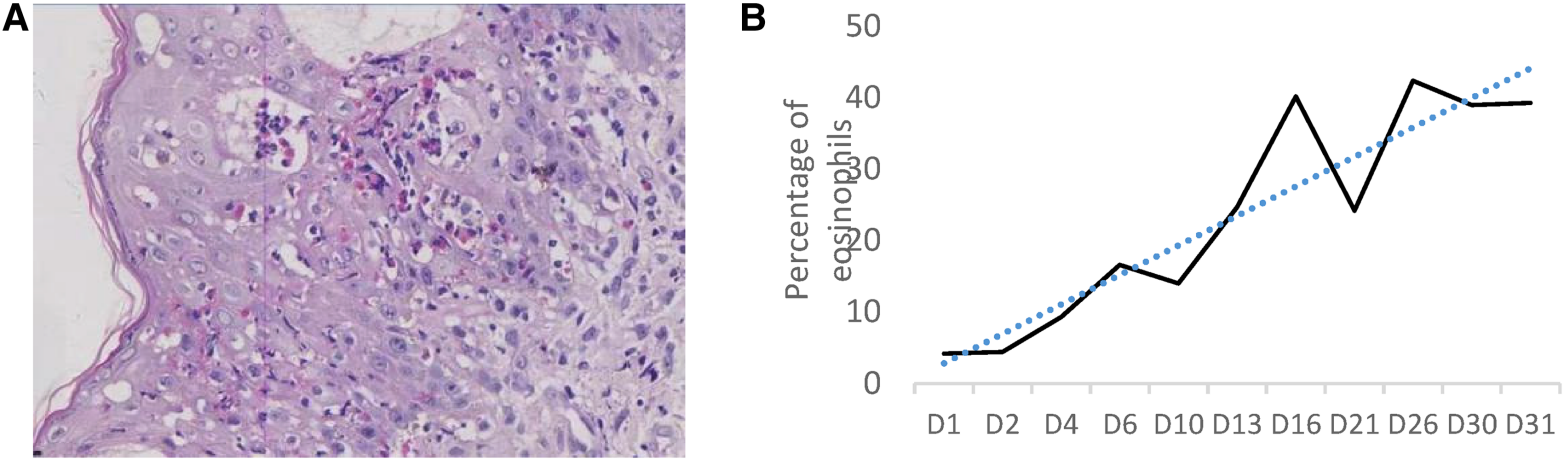
(**A**) Pathological findings of the skin biopsy of the lesion at the right thigh: eosinophilic spongiform edema of the lesioned skin, eosinophilic microabscesses in the focal epidermis, liquefaction of the basal layer, and eosinophilic infiltration in the superficial dermis; (**B**) trends in peripheral blood eosinophil percentage during hospitalization.

**Figure 3:**
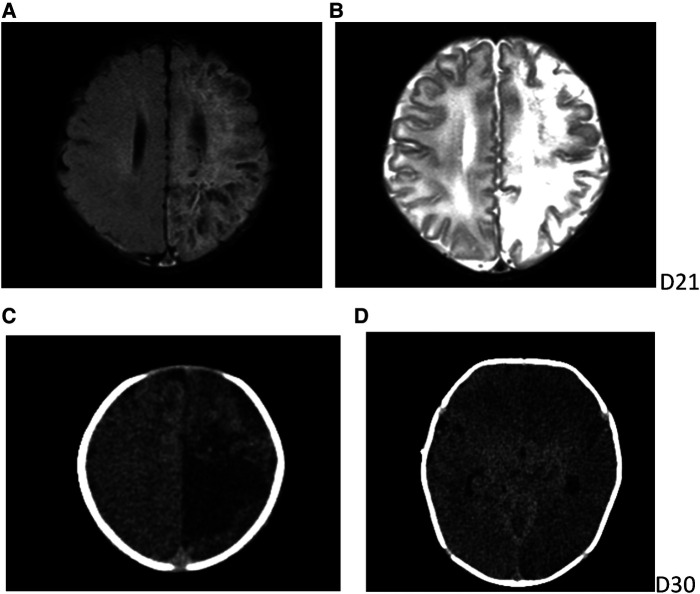
Cranial imaging findings. Routine brain MRI on day 21. (**A,B**) Diffuse multiple cystic and lamellar abnormal signal areas in the left frontal and parietal lobes with low signal in T1-weighted imaging and high signal in T2-weighted imaging, with speckled diffusion restriction within the lesion. Urgent cranial CT on day 30. (**C,D**) Diffuse hypodensity in the left cerebral hemisphere and bilateral cerebellum, with a large patchy hypodense area in the left frontoparietal lobe with a CT value of 10–15 HU and multiple speckled hyperintensities within it, a widened left ventricle and additional patchy hyperintensities in the left cerebellar hemisphere.

After admission, complete blood count, C-reactive protein and procalcitonin were normal; eosinophils were 4.2%, and viral spectrum screen and blood antinuclear antibody were negative. Herpes fluid culture was negative and repeated markers of infection were normal on day 3. New pustules were recurrent on the right knee and blood eosinophils increased to 16.6%; a skin biopsy of the thigh was taken on day 6 and the pathology reported eosinophilic spongiform edema of the lesioned skin, eosinophilic microabscesses in the focal epidermis, liquefaction of the basal layer, and eosinophilic infiltration in the superficial dermis after 10 days (Figure 2A). Blood eosinophils were consistently elevated, up to 42.4% on day 26 (Figure 2B). There were no abnormal findings on multiple cranial ultrasound examinations, but routine brain magnetic resonance imaging (MRI) on day 21 revealed diffuse multiple cystic and lamellar abnormal signal areas in the left frontal and parietal lobes with a low signal in T1-weighted imaging and a high signal in T2-weighted imaging, with speckled diffusion restriction within the lesion (Figures 3A,B), and no abnormalities in the sacrococcygeal MRI scan. The infant continued to have recurrent herpes, which worsened and fused into patches, followed by warty crusting and hyperpigmentation (Figures 1B–D). On day 30, the infant presented a marked decrease in milk intake, poor response, and apnea. An urgent cranial computed tomography (CT) scan found diffuse hypodensity in the left cerebral hemisphere and bilateral cerebellum, with a large patchy hypodense area in the left frontoparietal lobe with a CT value of 10–15 HU and multiple speckled hyperintensities within it, as well as a widened left ventricle and additional patchy hyperintensities in the left cerebellar hemisphere (Figures 3C,D).

#### 2.2.2 Genetic testing

Given the rarity of the case, and to clarify the potential molecular etiology of the case and to help determine the risk of the disease in the family, genetic testing at the chromosomal and genetic levels was performed with the consent of the patient's parents.

##### 2.2.2.1 Chromosomal copy number variation (CNV-Seq plus) and cytological karyotype detection

**Figure 4:**
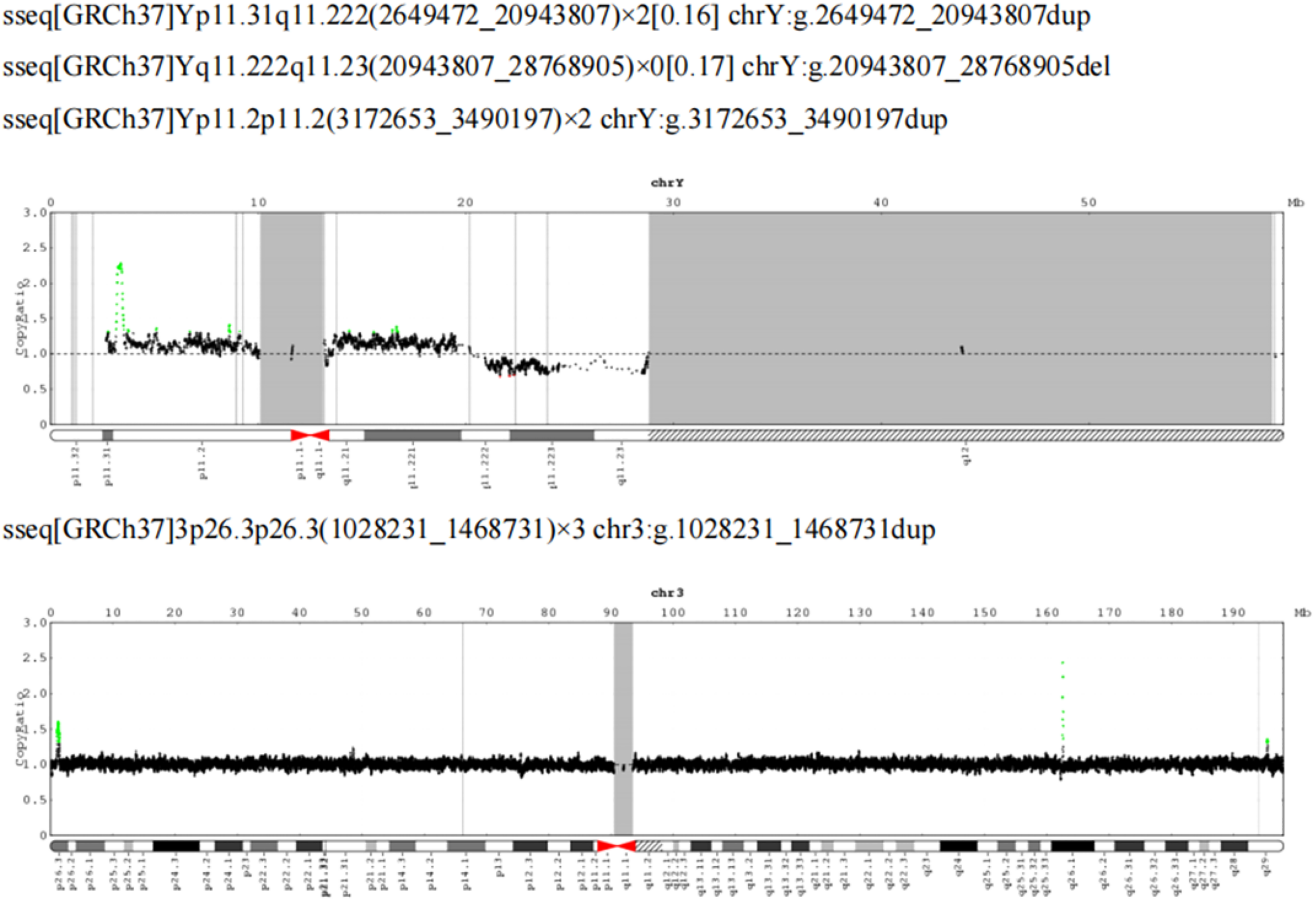
CNV-Seq plus: 18.29 Mb mosaicism repeats in the Yp11.31q11.222 region (16%), 7.83 Mb mosaicism deletions in the Yq11.222q11.23 region (17%), 317.54 kb repeats in the Yp11.2p11.2 region (copy number: 2) and 3p26.3p26.3 region, with approximately 440.50 kb duplication (copy number 3).

Peripheral blood was taken for CNV-Seq detection. No triploidy was detected; however, approximately 18.29 Mb mosaicism repeats were detected in the Yp11.31q11.222 region (16%), approximately 7.83 Mb mosaicism deletions in the Yq11.222q11.23 region (17%), approximately 317.54 kb repeats in the Yp11.2p11.2 region (copy number: 2) and 3p26.3p26.3 region with approximately 440.50 kb duplication (copy number 3) (Figure 4). To clarify the existence of mosaicism in this family, blood was collected from the second twin and parents for a karyotype analysis, which showed normal parental karyotype and the brother of 46,X,del (Y) (q11.23) [7]/46,XY [93] with a mosaicism rate of 7%.

##### 2.2.2.2 Whole exome sequencing and multiplex ligation-dependent probe amplification detection

The entire sequence of the coding region of the IKBKG gene was examined by whole exome sequencing (WES), and no pathogenic minor variants were found. Considering the existence of pseudogenes in IKBKG, WES may not be able to distinguish pseudogenes and detect variants. Then multiplex ligation-dependent probe amplification (MLPA) testing was performed and no large deletion repeat variants in large segments of the IKBKG gene were found; multiplex PCR amplification testing did not find deletion variants in exons 4–10 of the IKBKG gene.

### 2.3 Diagnosis and differential diagnosis

The main diagnosis of male neonatal IP complicated by severe cerebrovascular lesions was based on the following: (1) recurrent characteristic skin erythema and herpes, warty crusts, and hyperpigmentation as well as significant elevation of blood eosinophils, eosinophilic spongiform edema, and eosinophilic infiltration of the lesioned skin; and (2) abnormal signal areas in the frontal and parietal lobes on brain MRI in the asymptomatic phase and emergency cranial CT after the onset of neurological symptoms, which revealed abnormal changes in the left abnormal changes in the cerebral hemispheres and bilateral cerebellum, thought to be cerebral infarction combined with multiple foci of hemorrhage. Early differentiation of the disease from neonatal impetigo, herpes simplex virus infection, congenital syphilis, and herpetic epidermolysis bullosa was ruled out using normal infection markers repeated after admission and negative herpes fluid cultures, viral screening, and syphilis tests.

### 2.4 Treatment

Cefuroxime was administered intravenously after admission and discontinued on day 10 after a bacterial infection was ruled out and there was a high suspicion of IP. The characteristic skin lesions recurred and a potassium permanganate bath and topical application of mupirocin or fusidic acid were given for 1 month. Milk was initiated on day 2 and reached full oral enteral feeding on day 6. When the patient developed neurological symptoms and hypotension on day 30, he was treated with mechanical ventilation, vasoactive drugs (dopamine, dobutamine, epinephrine), and total parenteral nutrition for 2 days.

### 2.5 Outcome and follow-up

The typical skin lesions of IP recurring in different areas during the infant's hospitalization improved significantly with medicated baths and topical applications, without secondary infection. After 2 days of treatment with ventilators and vasoactive drugs, the infant died on the same day after his parents chose hospice care in view of the poor prognosis. The second twin had a birth weight of 2,260 g and was discharged from our hospital after 10 days of hospitalization for preterm low birth weight with no IP-related manifestations. He is now 1 year old and has normal growth and development at several follow-up visits.

## 3 Discussion and conclusions

We diagnosed a case of male neonatal IP complicated with severe cerebrovascular lesions from the typical clinical presentation, skin pathology, and brain imaging findings. This case was unique in that the severe skin and cerebrovascular lesions were present in only one male monozygotic twin, while the second twin was completely unaffected. This condition has not been reported in the literature.

IP is a rare neuroectodermal dysplasia disease caused by mutations in the IKBKG gene, usually with characteristic skin lesions evolving in a sequence of four stages that appear along the Blaschko line: the erythema and blisters, verrucous lesions, hyperpigmentation, and hypopigmentation (4). There may be more than one lesion type in different stages and the location of the lesions may vary from stage to stage (1). The diagnosis of IP is clear in this case, with typical erythema and blisters at birth, as well as recurrent blisters, warty crusts, and hyperpigmentation at different sites during the course of the disease, combined with significantly elevated blood eosinophils and skin pathology findings. Of cases of IP, 88% may present with elevated blood eosinophils, and the percentage may be as high as 65%, especially in stages I and II. This may be due to a mutation in the IKBKG gene that weakens nuclear factor kappa B inhibition, the susceptibility of mutant cells to apoptosis, and the overexpression of the eosinophil-selective chemokine eotaxin, leading to an excessive inflammatory response causing perivascular and intravascular infiltration, vascular occlusion, and ischemia (5, 6). In the infant, the eosinophils increased gradually after birth, reaching a peak of 40% after 2 weeks, and remained at a high level, which may be closely related to the child's recurrent skin lesions and subsequent cerebrovascular lesions, and is a cause for concern.

Abnormalities in the central nervous system occur in 25%–50% of patients with IP and are most common in the first year of life and more likely in male infants (7, 8). Developmental cerebral microangiopathy caused by IKBKG gene variants that inactivate IKBKG and upstream transforming growth factor-β-activated kinase disruption may lead to transient cerebral ischemia or hemorrhagic stroke (9–11). Neurological manifestations can be diverse, and cerebrovascular accidents may underlie neurological manifestations in the neonatal period. Epilepsy is the most common symptom, present in 13%–25% of patients with IP, and most epilepsy occurs within the first week of life (12). Routine MRI in this case already revealed signs of local inflammation, ischemia, and hemorrhage in the cerebral vasculature; however, a further electroencephalogram and consultation with a neurologist was overlooked due to the lack of neurological symptoms and the lack of awareness of this rare disease, resulting in serious neurological symptoms and severe cerebral infarction and hemorrhage. Therefore, for surviving male infants with IP, even if they are temporarily free of neurological symptoms, high vigilance is needed to actively perform imaging and brain electrophysiology examinations and promptly consult a pediatric neurologist. Due to the anti-inflammatory effect of glucocorticoids, there have been several reports of their use in the treatment of combined cerebrovascular lesions; however, its efficacy is difficult to prove, as the lesions may disappear on their own without treatment (13).

The currently known IP causative gene is the IKBKG, located at Xq28, approximately 33 kb in length, containing 10 exons and 9 introns and encoding the IKBKG protein. It is a key protein in the nuclear factor kappa B signaling pathway, which is important for various key cellular biological processes such as cell proliferation, cell survival, cellular stress response, innate immunity, and inflammation (14). IP is X-linked dominant, and mutations in this gene can have different effects in males and females. Female patients have a wide range of phenotypes and can be associated with ocular, skeletal, and central nervous system damage, while male fetuses carrying the mutated gene may die *in utero* because they lack the IKBKG protein necessary for survival (15).

There are two 870 bp direct repeats in the IKBKG gene, called MER67B, one in intron3 and the other located downstream of IKBKG. Recombination between the MER67B repeats results in exons 4–10 of IKBKG missing, a common 11.7 kb deletion. Of the currently reported IP cases, 90% are caused by exon 4–10 deletions (16); however, there may also be point mutations, indel mutations, and other possibilities in the IKBKG gene. A highly homologous pseudogene, IKBKGP1, exists in the 22 kb repeat region downstream of the IKBKG gene, with sequence orientation opposite to that of the IKBKG gene and highly similar to exons 3–10 of IKBKG. Because of the presence of the pseudogene, conventional high-throughput sequencing cannot sequence the entire gene, and MLPA is usually used to detect the common 11.7 kb deletion; therefore, failing to find a clear genetic mutation in patients with IP is not uncommon. Despite the large number of male fetuses with IP that die *in utero*, a few cases of male IP survival have been reported, with a currently presumed male-to-female ratio of 1:20 (2). Male survival is commonly thought to be associated with one of two mechanisms: karyotype 47,XXY and somatic mosaicism (17). In addition to the difficulty of detecting mutations beyond the classic 11.7 kb deletion, somatic mosaicism can result in the failure to detect a pathogenic mutation in peripheral blood, in which case molecular genetic testing of a tissue sample (e.g., skin from an affected site, sperm) may be needed (18). For these reasons, the rate of positive genetic testing in men with IP is low, with one review of the male IP literature finding that 58% of men tested negative for genes (19).

This case is considered to be caused by somatic mosaicism because neither of the parents had a family history of IP and the infant's CNV-Seq Plus results were suggestive of somatic mosaicism. The second twin was phenotypically normal, but a chromosome culture of his peripheral blood cells revealed 46,X,del (Y) (q11.23) [7]/46, XY [93], indicating a 7% Y chromosome chimerism. Given that the Y chromosome variant primarily affects the male reproductive system and that the twins were newborns, it was not possible to observe any effects on the reproductive system. Consequently, it can be concluded that the IP phenotype of the infants is not related to the Y chromosome chimerism. Although cases of monozygotic twins have been reported to develop IP alone or simultaneously, they have all been in female twins (20). This has not been reported in cases where one male monozygotic twin is affected and the other with the same genetic material is unaffected. This phenotypic inconsistency may be related to the different levels of somatic cell mosaicism in twins (20).

A limitation of this paper is that although high-throughput sequencing, first-generation sequencing, MLPA large fragment detection, and multiplex PCR for exon 4–10 variants of the IKBKG gene were used, no deletion variants or point mutations were found. Hence, the presence of an 11.7 kb deletion mutation was ruled out in this case; a definitive molecular diagnosis was ultimately not obtained. To accurately detect mutations in similar male infants with IP, samples should be collected from as many affected sites as possible to improve detection rates, and third-generation sequencing and single-tube long fragment read can be used to eliminate the effect of pseudogenes. Furthermore, it is possible that the IKBKG gene may not be expressed due to imprinting disorders. However, we were unable to confirm this possibility at the expression level due to the unavailability of a valid specimen from the child's death and technical limitations.

In conclusion, this paper reports an extremely rare case of neonatal IP with the onset of one male identical twin whose molecular diagnosis was challenging and phenotypic inconsistency may have been related to the level of somatic chimerism. The poor prognosis of combined severe cerebrovascular lesions suggests the need to raise awareness of the disease and to actively complete relevant investigations, and to explore potential prevention and treatment options.

## Data Availability

The original contributions presented in the study are included in the article/**Supplementary Material**, further inquiries can be directed to the corresponding author.
