# Supplementary figures and images for: A case report of neonatal incontinentia pigmenti complicated by severe cerebrovascular lesions in one of the male monozygotic twins
Source: Front Pediatr. 2024 May 21;12:1338054. doi: 10.3389/fped.2024.1338054 (PMC11144854; doi:10.3389/fped.2024.1338054)

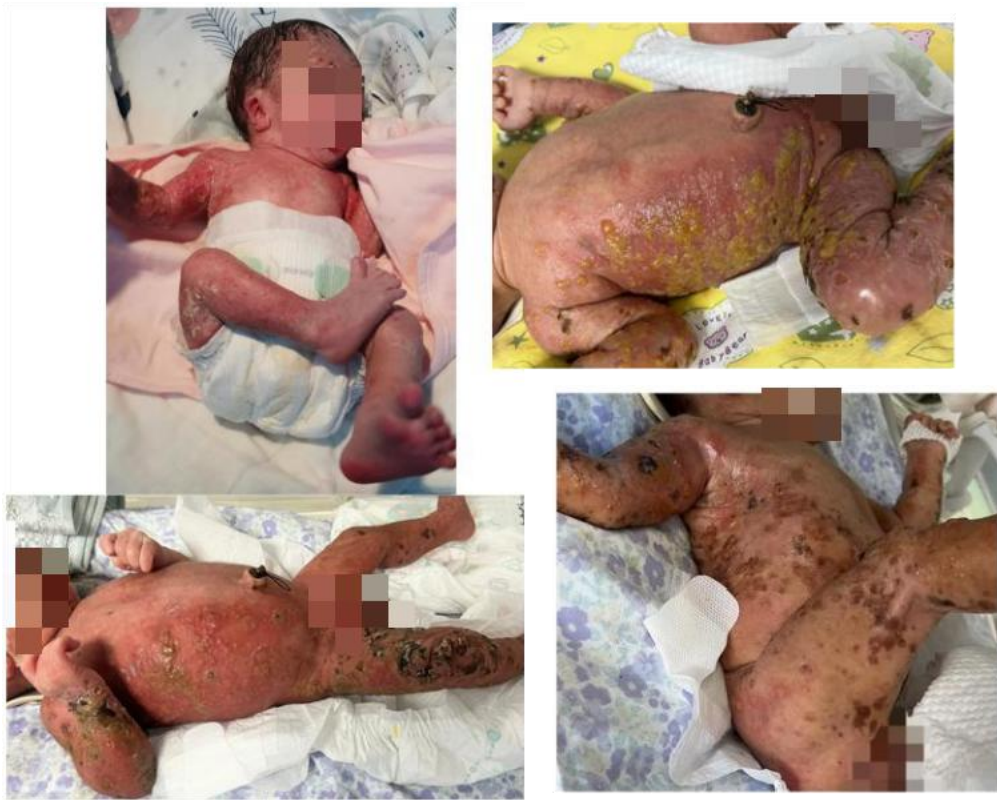

**Manifestations of skin lesions at different times**

Supplement: Supplementary file 4 [file Image1.pdf]
